# Pilonidal Sinus of the Glans Penis Associated with *Actinomyces* Case Reports and Review of Literature

**DOI:** 10.1100/tsw.2004.188

**Published:** 2004-10-22

**Authors:** Shylashree Chikkamuniyappa, Jaime Furman, Rolf Sjuve Scott

**Affiliations:** Department of Pathology, University of Texas Health Science Center at San Antonio, TX, USA

**Keywords:** prepuce, penis, pilonidal sinus

## Abstract

Pilonidal sinus is a well-recognized condition that occurs most commonly in the sacrococcygeal area of younger men. It is hypothesized to be an acquired chronic inflammation condition due mainly to hair trapped beneath the surface. A pilonidal sinus in the sacrococcygeal region is associated with recurrent infection, abscess formation, cellulitis, fistulae, and rarely, squamous cell carcinoma. A pilonidal sinus of the penis is a rare entity. The association of a penile pilonidal cyst and Actinomyces is even more uncommon with only three cases reported previously. Two cases of pilonidal sinus are reported in this paper. One of the cases was associated with actinomycosis. Pilonidal sinus of the penis should be considered in the clinical and pathological differential diagnosis and has to be distinguished from balanoposthitis, epidermal cyst, and carcinoma. The knowledge about possible association with actinomycosis is important to ensure early treatment.

## INTRODUCTION

A pilonidal cyst or sinus is an abscess or a chronic draining sinus usually containing hairs. The term was first coined by Hodges in 1880[1] with “pilus” meaning hair and “nidal” meaning nest in Latin. It has been reported in other areas, including the umbilicus[2], interdigital cleft (“Barber’s disease”)[3], perineum, amputation stump, and as a cyst in the breast[4]. A pilonidal sinus in the sacrococcygeal region may be associated with recurrent infection, abscess formation, cellulitis, fistulae, and rarely, squamous cell carcinoma[5,6]. A pilonidal sinus of the penis is a rare entity, with very few reported cases[7]. They clinically present as a classic case of inflammation with pain, local infection, and redness, but may also show chronic ulceration or a draining sinus or abscess formation.

In this paper, we report two cases affecting the penis and also review the literature. One of the cases was associated with actinomycosis.

## CASE REPORTS

The first patient is a paranoid schizophrenic, uncircumcised, 42-year-old Hispanic male who presented with a 2-week history of penile swelling and phimosis, which did not resolve with antibiotics (Amoxicillin, 500 mg bid × 10 days and Ceftriaxone, 1 g). He also has an indurated fistula-in-ano draining midline abscess anterior to the anus. On examination, a symmetrically enlarged, phimotic penis with signs of local inflammation was seen. A nonfluctuating, firm mass was palpated under the foreskin. There was bilateral inguinal lymphadenopathy. There was no drainage from the urethral meatus. Notable was his white cell count of $15.2 \times 10^3$/ul with 65.7% neutrophils. An elective circumcision, penile biopsy, and rectal examination under anesthesia with sinus tract repair were performed.

The second patient is a 22-year-old, previously healthy, uncircumcised Hispanic male. He presented with a several week–long history of phimosis with a foreskin nodule that was draining caseous fluid. Clinically, an epidermal inclusion cyst was suspected. Circumcision with radical excision of the nodule was performed.

## PATHOLOGIC FINDINGS

### Gross Pathology

Patient 1: The circumcised foreskin measured 6.5 × 4.5 × 2 cm. The specimen was bisected and showed a dorsal, firm, white diffuse, ill-defined mass with central hemorrhagic areas and small cysts. No sinuses were grossly visible.

Patient 2: The circumcised foreskin measured 6.0 × 3.5 × 2.0 cm. The skin surface on the dorsal aspect showed a gray-yellow, slightly raised nodule measuring 0.3 × 0.3 cm. The sectioned specimen showed a gray-white, ill-defined mass with no apparent hemorrhage measuring 1.7 × 1.2 × 1.5 cm.

### Microscopic Pathology

Representative sections from both specimens of the center, proximal, distal, and lateral aspect of the lesions showed multiple abscesses with acute and chronic inflammation. There were areas of reactive fibrosis and necrosis. Hairs were present in the coronal sulcus and hair shafts embedded in the subcutaneous tissue were surrounded by a foreign-body giant cell reaction (Fig. 1a,b). The specimen from patient 2 showed hairs with abscess formation, foreign-body giant cell reaction, and also showed conspicuous sulfur granules consistent with *Actinomyces* species (Fig. 2). There was no evidence of malignancy with normal stratified squamous epithelium.

## COMMENT

Pilonidal sinus of the penis is a rarely reported entity in uncircumcised men, the most common site being the region around the corona involving the foreskin. It was first reported by Bervar et al.[8] in 1968. Thirteen papers have previously been published (age range: 21–59, mean age: 31)[9,10,11,12,13,14,15, 16,17,18,19,20,21]. None of the reported cases was circumcised. The patients had had symptoms with swelling and/or drainage for 1 week up to 4 years. An unusual presentation was erectile dysfunction[17]. Histological examination ranged from a small abscess formation with hair fragments and a foreign-body reaction to a re-epithelialized sinus tract extending along the entire shaft. The most common location was the dorsal aspect of the foreskin[7]. In three of the cases, a carcinoma was suspected[10,14,19].

Interestingly, in three of the cases, the color of the hairs found in the sinus did not match that of the patients own hair color[12,13,15]. All the reported cases healed after circumcision with no recurrence. Cases associated with *Actinomyces*, including the present, ranged in age from 21–53 years of age[7,20,21].

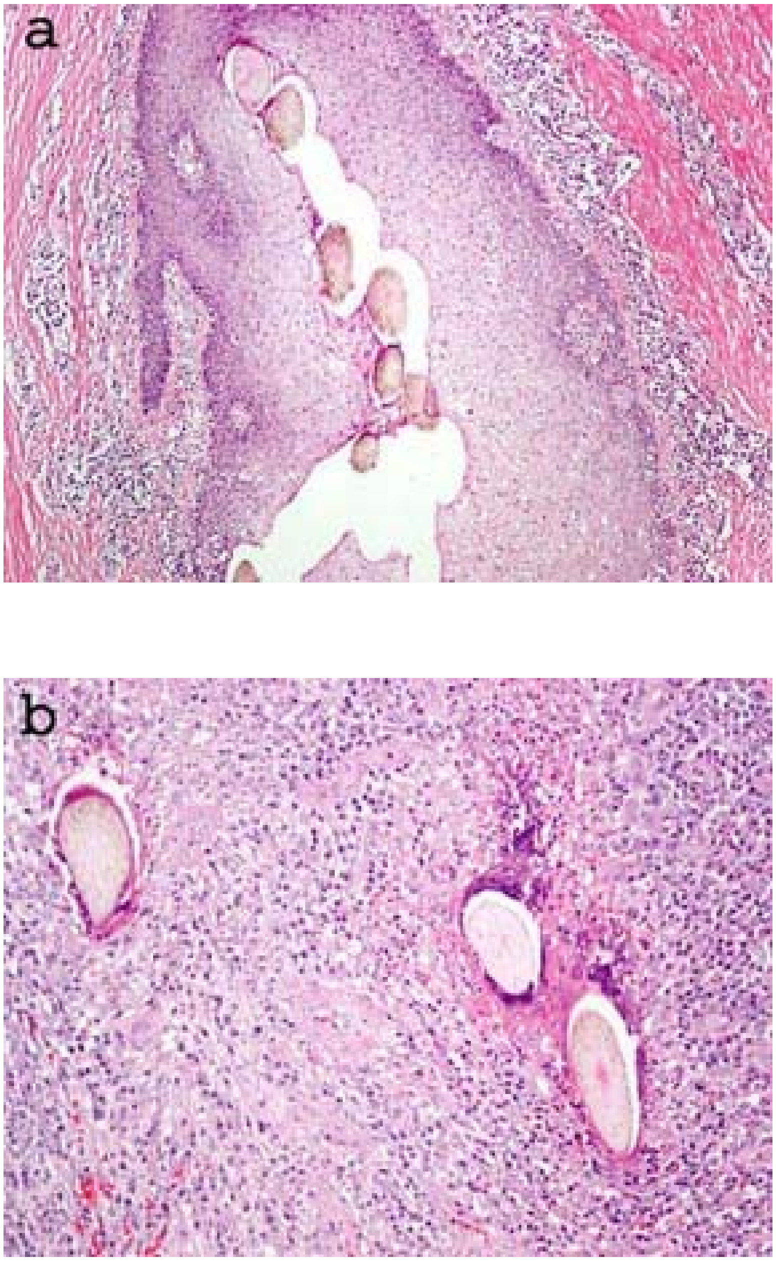

**FIGURE 1.** (a) Hair shafts in the coronal sulcus, 100×; (b) hair shaft with acute and chronic inflammation and foreign-body giant cell reaction, 100×.

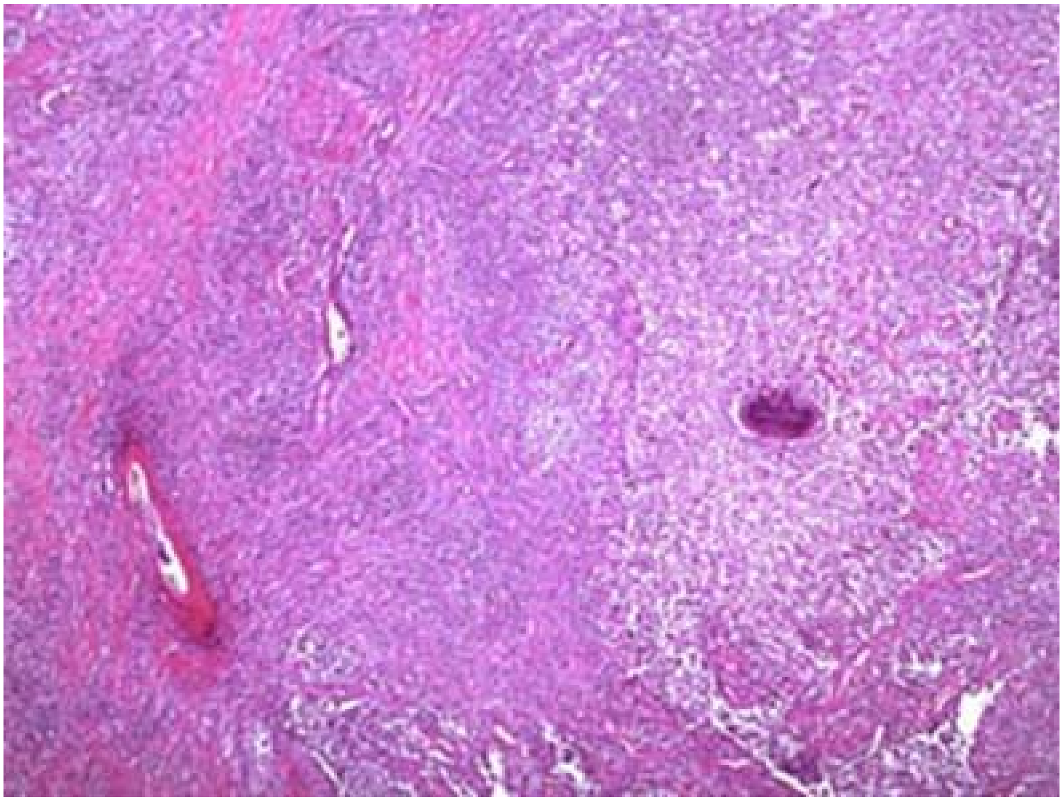

**FIGURE 2.** Sulfur granule consistent with actinomycosis and hair shaft with acute inflammation, 40×.

Pilonidal sinuses in general are currently accepted as being an acquired pathology[22,23] involving the midline pits in the natal cleft. The sinuses develop when a dead or distorted hair is pushed by mechanical forces into tiny abrasions or scars in the skin, forming a one-ended tunnel where bacteria and necrotic debri may collect. A cyst is formed, which becomes edematous and ruptures. A foreign-body reaction results in microabscesses from the keratin and pus. A sinus lined by squamous epithelium may form to drain the suppuration. For pilonidal sinus to occur in the penis, it is hypothesized that the coronal sulcus acts as a cleft where hair may accumulate from surrounding hairy areas of the patient or possible partner. The hairs are then driven into the shaft and prepuce by the mechanical forces and the rolling movement at the junction of glans penis and the uncircumcised prepuce[18]. There have been three previous cases reported of the association of penile pilonidal sinus with actinomycosis[7,20,21]. This branching and generally low, virulent, Gram-positive organism is a normal commensal of the gastrointestinal tract and causes recurrent abscesses or nonhealing ulcers and may mimic a neoplastic lesion. Previous reports have described actinomycosis in other locations in the genital tract of males[24,25,26]. The infection is rarely related to systemic disease in immunocompromised hosts. Malignant transformation of pilonidal cysts is an extremely rare complication with squamous cell carcinoma being the most commonly diagnosed[5,6]. Squamous cell carcinoma associated with pilonidal cysts of the penis has not been reported, but the possibility should be kept in mind by both clinician and pathologist.

Simple excision with primary closure or healing by granulation tissue and sinus tract excision is usually the treatment for pilonidal sinus. If pus is available, it should be sent for culture and Gram stain. Long-term penicillin is prescribed after cultures and antibiotic susceptibilities to prevent sepsis. This is followed by circumcision to treat the underlying phimosis.

In conclusion, pilonidal sinus should be considered an important differential diagnosis in a case of penile swelling, nonhealing ulcer, or phimosis. Awareness of the association of pilonidal cysts of the penis and actinomyces among both clinicians and pathologists is necessary to come to an early diagnosis and treatment. Surgical excision of the pilonidal sinus and in the case of concomitant infection with actinomycosis, a prolonged treatment with antibiotics, is important to avoid morbidity or systemic infection.
